# Tailoring Release Protocols to Individual Species and Sites: One Size Does Not Fit All

**DOI:** 10.1371/journal.pone.0099753

**Published:** 2014-06-25

**Authors:** Katherine E. Moseby, Brydie M. Hill, Tyrone H. Lavery

**Affiliations:** 1 The University of Adelaide, North Terrace, Adelaide, Australia; 2 Arid Recovery, Roxby Downs, Australia; 3 School of Biological Sciences, The University of Queensland, St Lucia, Australia; University of KwaZulu-Natal, South Africa

## Abstract

Reintroduction programs for threatened species often include elaborate release strategies designed to improve success, but their advantages are rarely tested scientifically. We used a set of four experiments to demonstrate that the influence of release strategies on short-term reintroduction outcomes is related to both intrinsic and extrinsic factors. We compared different reintroduction strategies for three mammal species in an arid environment where exotic mammalian predators were removed. Wild greater stick-nest rats selected vegetation shelter sites with greater structural density than captive-bred rats, travelled further from the release site and experienced lower rates of mortality. In comparison, there was no difference in mortality or movement between wild and captive-bred greater bilbies. Burrowing bettongs and greater bilbies were also subjected to immediate and delayed release strategies and whilst no difference was detected in bilbies, bettongs that were subjected to delayed releases lost less weight and took less time to establish burrows than those that were immediately released. Interspecific differences in treatment response were attributed to predation risk, the nature of the release site, and behavioural traits such as shelter investment and sociality. Our varied results highlight the inadequacies of review articles focusing on optimum release protocols due to their attempt to generalise across species and release sites. We provide an example of a predictive model to guide future release strategy experimentation that recognises the range of intrinsic and extrinsic factors influencing reintroduction outcomes. We encourage researchers to treat programs experimentally, identify individual site and species characters that may influence release strategies and record data on movements, mortality, weight dynamics, and settling times and distances. The inherent issues of small sample size and low statistical power that plague most reintroduction experiments suggests there is also a need for increased standardisation and publication of data sets to enable appropriate meta-analyses to occur.

## Introduction

Species reintroductions are now widely used in conservation programs throughout the world and are defined by the IUCN [1] as “the intentional movement and release of an organism inside its indigenous range from which it has disappeared”. Yet, although reintroductions are widely practised, the successes of reintroduction programs (here defined as the establishment of a viable, self sustaining population) continue to vary greatly for different species under different scenarios. Bajomi [2] summarised the results of five global reintroduction reviews that reported between 11 and 62% of reintroduction programs as successful. At a global scale, factors cited as influencing reintroduction success include predation risk, habitat quality, release site range relative to historical area and the size of release populations [3]–[5]. Predation by introduced mammalian predators is the most significant cause of reintroduction failure in Australia [6]–[8]. As a result of this threat, more than half of 200 reviewed reintroductions in Australia have been into areas where exotic predators are excluded (82) or heavily controlled (30) [7]. Similarly, in New Zealand more than half (60) of the 96 bat, reptile and amphibian reintroductions have been onto islands where exotic mammals were absent or controlled [9].

Practitioners often attempt to further improve reintroduction success by varying release protocols. Release protocols include components such as the choice of release site, the source of animals for release and the pre and post release support provided [10]. Source populations can be obtained from the wild or from captive-bred stock. Post release support can include the provision of food, shelter and/or water [11]. This style of release strategy also often employs an initial period of on-site containment designed to reduce the large scale post release movements [12], [13] that are considered to increase mortality and prevent establishment of cohesive populations [14]–[16]. Protocols that include the provision of these forms of support have been generally termed ‘soft’ releases in the literature with the belief that a period of containment on-site may also give animals time to adjust to their new surrounding and minimise mortality [15] and stress [17]. However, recent studies have found that this style of release can also be detrimental to long-term survival of some species [18]. The terms ‘soft’ and ‘hard’ release are misleading as they imply benefits or detriments to the reintroduced species that are rarely tested experimentally (e.g. [18]). Thus we herein refer to hard and soft release strategies as immediate and delayed releases, where our delayed releases also include supportive measures such as the provision of food and shelter.

In Australia, some researchers believe delayed release strategies improve mammalian reintroduction success [19]–[21]. Conversely, other studies have found no difference in mortality, movement or condition of animals released with or without supportive measures [11], [22], [23]. The results of global review papers on the success of reintroductions using different release strategies have been inconsistent and contradictory. Fischer and Lindenmayer [6] found that globally, reintroductions using wild stock were more successful than those using captive-bred animals. However, Wolf et al. [5], [6] and Short [7] reviewed Australian and/or North American reintroductions and found no difference in success between mammal reintroductions using captive or wild stock. Fischer and Lindenmayer [6] also presented evidence to show that the failure rate of reintroductions is reduced if supportive measures are undertaken but Short [7] concluded immediate releases in Australia were typically more successful. Griffith et al. [3] and Wolf et al. [4], [5] found no significant differences in survival between delayed and immediate releases of mammals and birds in North America and Australia.

The inconsistencies in the reported relationships between different release strategies and post release survival are likely due to the interplay between site conditions, predation pressure and behavioural and life history traits of study species. In certain conditions, delayed releases or the provision of food or water might improve short-term reintroduction outcomes by reducing movement or starvation, and/or containing animals within a high-intensity predator management zone (e.g. [15], [24]). Social, sedentary species that invest heavily in building warrens or shelter sites may further benefit from delayed releases or provision of artificial shelters, giving them time to form social groups and construct shelters (e.g. [16]). However, delayed releases of wild individuals could increase stress in certain species by prolonging time in captivity [1]. Predation risk can also influence the appropriateness of release strategies. Using predator-aware stock in areas of high predation risk could theoretically improve the chance of post release survival, although this has been little tested (see [25] for one example). However, any differences in anti-predator behaviour between captive and wild source populations may have little influence on post release survival when predation pressure is low. In these situations, immediate releases may represent a cheaper, simpler and more efficient method of release.

Overall, the utility of many review papers on reintroduction protocols is limited because they report the average successes of reintroductions using very broad groupings of release strategies and do not take into account site and species-specific characters. These do not inform practitioners of the circumstances under which a particular release strategy is likely to succeed, and as such, they fail to guide future releases on the best strategy to use. Furthermore, comparative analyses can be misleading. Short [7] found that in Australian reintroductions, small release group sizes were more likely to be successful than large group sizes. However, this review did not consider the confounding factor that small groups were most often released into predator-free enclosures.

Many researchers have called for reintroductions to be conducted as experiments to improve the science of reintroduction biology [1], [26], [27]. However, reintroduction practitioners rarely take advantage of the opportunities to experimentally test the influences of release protocol on the success of a reintroduction program. Instead, they generally rely on past experience, intuition, anthropomorphism or the precautionary principle (e.g. [28]), rather than science [29]. Short et al. [30] found that most macropod reintroductions in Australia used a delayed release despite the lack of evidence that it is beneficial. Releases rarely use control groups, and seldom collect and report data on movements, weight dynamics, shelter site establishment, and short-term survival that may affect outcomes. These shortfalls in experimentation may be partly a result of most reintroductions targeting highly endangered species. The numbers of animals available for reintroductions are simply too limited, and this reduces the statistical power of single experiments and the conclusions that can be drawn from their results.

We conducted a set of four controlled experiments on reintroductions of arid zone mammals to a fenced reserve in Australia to demonstrate that release strategies need to be individually tailored to suit particular species and locations. We used three species, each with different behavioural and social traits, ranging behaviour, and shelter site fidelity. Data on short-term post release behaviour, mortality, body condition and movement were compared for three species, wild and captive source populations, and immediate vs delayed releases. The results of these experiments and other published studies are used to describe how intrinsic and extrinsic factors may influence appropriate release strategies. More importantly, results are used to suggest a framework and predictive model for future experiments and reviews that examine the influence of release strategies on reintroduction outcomes.

## Materials and Methods

### Ethics statement

Reintroductions were conducted under ethics approval from the Wildlife Ethics Committee, South Australia and permits from the South Australian Department for Environment and Natural Resources. All efforts were made to minimise suffering of animals during trapping, handling and reintroduction.

### Study site

Established in 1997, the Arid Recovery Reserve (30°29′S, 136°53′E) is a 123 km^2^ fenced exclosure situated 20 km north of Roxby Downs in arid South Australia. A 1.8 m high, wire netting fence with a curved overhang excludes the exotic European rabbit (*Oryctolagus cuniculus*), feral cat (*Felis catus*) and red fox (*Vulpes vulpes*) from a 60 km^2^ area [31]. Internal fencing separates the reserve into four paddocks (Main, First, Second and Northern Paddocks) and regular spoor monitoring is conducted to confirm that exotic mammals remain excluded. The dominant landforms within the reserve are longitudinal orange sand dunes separated by 100 m to 1 km wide clay interdunal swales. The climate is arid and rainfall is aseasonal, failing to reach its long-term average rainfall of 166 mm in 60% of years [32].

Four locally extinct nationally threatened mammal species, the greater stick-nest rat (*Leporillus conditor*), greater bilby (*Macrotis lagotis*), western barred bandicoot (*Perameles bougainville*) and burrowing bettong (*Bettongia lesueur*), were reintroduced to the 14 km^2^ Main Paddock of the Arid Recovery Reserve between 1999 and 2001 [33]. All of these species have been extinct on the mainland of South Australia for more than 50 years [34]–[36] due to a combination of predation from introduced foxes and cats, and habitat degradation from rabbits and domestic stock [34], [35], [37], [38].

### Reintroductions

This study outlines four experimental reintroductions of three of these species into the Northern Paddock of the reserve using a range of reintroduction strategies including wild vs captive-bred release groups and delayed vs immediate releases (Table 1). Small sample sizes prevented all three species from receiving all treatment combinations. Spoor counts suggested that very low densities of bilbies (<2 tracks per km of transect, estimated <5 animals) were present in the Northern Paddock at the time of the experiment, but no tracks were observed within 2 km of the release site (see [33] for method). No other reintroduced species were present.

**Table 1:** Reintroduction strategies and life history traits for three species reintroduced during the study at the Arid Recovery Reserve.

Species	Total	Range	Shelter Site	Sociality	Delayed Release		Immediate release	
	# Animals		Fidelity		Wild	Captive-bred	Wild	Captive-bred
Burrowing Bettong	14	Medium	High	High	8 (4M, 4F)	0	6 (3M, 3F)	0
Greater Bilby	12	High, transient	Low	Low	4 (2M, 2F)	4 (2M, 2F)	0	4 (2M, 2F)
Stick-nest	17	Low	High	Medium	0	0	7 (1M, 6F)	10 (5M, 5F)
Rat*	25						19 (10M, 9F)	6 (3M, 3F)

* = two separate releases.

The three reintroduced species used in this study differ in their behaviour and life history traits and were classified according to the categories of sociality, ranging behaviour and shelter site fidelity (Table 1). Burrowing bettongs are social animals that live in family groups and spend the daylight hours underground in multi-entranced warren systems [40]. They exhibit high burrow fidelity and at Arid Recovery they move on average 320 m from their burrows to their centres of activity [41]. In contrast, bilbies are mostly solitary, transient animals [39] that move burrows frequently and can shift their movements and diet in relation to seasonal conditions [42]. They are highly flexible [43] and can range over large areas with burrows up to 1 km apart [39]. Greater stick-nest rats are nocturnal, they construct communal nests from sticks and prefer areas of thick vegetation cover for nesting and foraging [35]. Stick-nest rats have well defined, relatively small home ranges and live in small family groups [44].

All captive-bred animals were reared at the Monarto Zoological Park near Adelaide, South Australia. Wild animals were caught from within the Main Paddock of the reserve, except for the first release of stick-nest rats where wild animals were obtained from Reevesby Island, South Australia. Animals subjected to delayed release strategies were placed in a 2 ha containment pen at the release site for three weeks. Prior to placing animals in the pen, one metre deep burrows were created, and dense piles of branches were constructed to provide immediate shelter to the animals. Supplementary food (rolled oats and peanut butter, and vegetables) and water was provided in the pen. After three weeks, holes were then cut in the pen and animals were allowed access to the full extent of the Northern Paddock. Animals that were immediately released were placed in the Northern Paddock, next to, but outside of, the containment pen.

We compared post release mortality, movement and body condition of animals in each treatment (Tables 1 and 2). When required, different release strategies were randomly assigned and where possible equal numbers of each sex were exposed to each treatment. All released animals were weighed, health checked and fitted with radio-transmitters before release. All animals were located after release by radiotracking, and weight and condition were assessed by recapture. The frequency of radiotracking and time of recapture after release varied between species due to differences in movement patterns and ease of capture between species. Statistical analyses were performed in SPSS version 18.0 (SPSS Inc., 2009) [45]. Individual methods and data analysis for the different experiments are outlined below.

**Table 2:** Radiocollared animals released into the Northern Paddock, their initial characteristics, date of access to Northern Paddock and release treatment.

Species	Date	Release Type	Sex	Weight	Condition	Pouch/Comments	Max KTBA (months)
Burrowing Bettong	15/10/2002	Immediate Wild	M	1668	Good		38
	15/10/2002	Immediate Wild	M	1539	Fair		7
	15/10/2002	Immediate Wild	M	1400	Good		8
	15/10/2002	Immediate Wild	M	1442	Poor		
	15/10/2002	Immediate Wild	F	1397	Good	Inactive	
	15/10/2002	Immediate Wild	F	1372	Good	Inactive	7
	15/10/2002	Immediate Wild	F	1460	Good	Inactive	
	15/10/2002	Immediate Wild	F	1446	Good	Inactive	15
	15/10/2002*	Delayed Wild	F	1255	Good	Inactive	
	15/10/2002*	Delayed Wild	F	1292	Good	Inactive	
	15/10/2002*	Delayed Wild	F	1528	Good	Inactive	16
	15/10/2002*	Delayed Wild	M	1382	Fair		6
	15/10/2002*	Delayed Wild	M	1643	Good		19
	15/10/2002*	Delayed Wild	M	1635	Good		3
Greater Bilby	29/4/2003	Immediate Wild	F	1159		Inactive+	21
	29/4/2003	Immediate Wild	F	952		Inactive+	
	29/4/2003	Immediate Wild	M	1194			
	29/4/2003	Immediate Wild	M	1088			
	8/4/2003	Immediate Captive	F	958		Inactive+	
	8/4/2003	Immediate Captive	F	1084		Inactive+	
	8/4/2003	Immediate Captive	M	960			
	8/4/2003	Immediate Captive	M	958			
	8/4/2003*	Delayed Captive	F	1040		Inactive+	
	8/4/2003*	Delayed Captive	F	990		Inactive+	
	8/4/2003*	Delayed Captive	M	1265			
	8/4/2003*	Delayed Captive	M	1030			
Stick-nest Rat	2/7/2003	Immediate Captive	M	290	Excellent		
	2/7/2003	Immediate Captive	M	362	Excellent		
	2/7/2003	Immediate Captive	F	283	Excellent		
	2/7/2003	Immediate Captive	F	363	Excellent		
	2/7/2003	Immediate Captive	M	359	Excellent		
	2/7/2003	Immediate Captive	F	314	Excellent		
	2/7/2003	Immediate Captive	F	309	Excellent		
	2/7/2003	Immediate Captive	F	360	Excellent		
	2/7/2003	Immediate Captive	M	326	Excellent		
	2/7/2003	Immediate Captive	M	385	Excellent		
	5/7/2003	Immediate Wild	M	330	Good		
	5/7/2003	Immediate Wild	F	120	Fair	Subadult	
	5/7/2003	Immediate Wild	F	130	Fair	Subadult	
	14/7/2003	Immediate Wild	F	165	Good		
	16/7/2003	Immediate Wild	F	244	Good		
	24/7/2003	Immediate Wild	F	275	Poor		
	27/7/2003	Immediate Wild	M	115	Fair	Subadult	26

For individuals that were opportunistically recaptured after completion of the study, the maximum time Known to Be Alive (KTBA) is included.

* = Date of translocation to the on-site containment pen, access to the rest of Northern Paddock occurred three weeks later.

+ = All females had pouch young when checked 7–9 weeks after release.

#### Burrowing bettongs – immediate vs delayed

On 15 October 2002, 14 bettongs were transferred to the Northern Paddock from the Main Paddock. Six were placed into the containment pen and eight were released immediately into the Northern Paddock (Table 2). Bettongs were radiotracked daily in the first week and every second day for a further three weeks. Delayed release animals were again located daily for the first week after access was allowed to the Northern Paddock, then every second day for a further three weeks.

Immediate release bettongs were recaptured at two and four weeks post release whilst delayed release bettongs were recaptured two weeks after being placed in the containment pen and then two and four weeks after the release pen was opened. Immediate and delayed release bettong weights were analysed using randomisation tests and a linear mixed model with release method and time as factors (using a compound symmetry correlation structure). Changes in weight measurements were compared over time using measurements from the beginning of the study, and at two and four weeks after access to the Northern Paddock. Some bettongs were also recaptured incidentally during trapping programs within the Northern Paddock over the following three years and their longevity recorded.

The daily movement of bettongs was calculated as the distance between consecutive diurnal locations. Once a bettong was recorded in the same diurnal location for two consecutive sampling days it rarely moved and it was assumed that the animal had established burrow fidelity. For animals with delayed releases, the time taken until burrow fidelity and the distance moved on the first night of release was recorded once they had left the containment pen. The time until burrow establishment in the Northern Paddock and the distance of the established burrow from the release pen were compared between immediate and delayed release animals using Student t tests and randomisation tests. Data were first tested for normality using the Shapiro Wilk test that is more appropriate for small sample sizes than the Kolmogorov Smirnov test. Data that were normally distributed were analysed using independent samples Student t tests. Levene's test for equality of variances was used and if significant then equal variances were not assumed. Data that were not normally distributed were transformed using ln+1 or compared using nonparametric Mann Whitney U tests. The *P*-values estimated using ANOVAs and student t-tests are based on the assumption that the data sample size is large enough such that it conforms to a normal distribution. Given our small sample sizes we were concerned these asymptotic methods may fail to produce reliable results. We thus supported these analyses by using randomisation tests [46].

#### Greater bilbies – wild vs captive, immediate vs delayed

On 8 April 2003, eight captive-bred bilbies were transferred to the Northern Paddock, four animals (2M, 2F) were immediately released and four delayed release bilbies (2M, 2F) were placed into the containment pen (Table 1). Delayed release animals were allowed access to the Northern Paddock on 29 April 2003 (following three weeks within the containment pen). On the same day, an additional four wild-born bilbies (2M, 2F) were translocated from the Main Paddock and immediately released to the Northern Paddock (Table 2). All bilbies were radiotracked daily for the first 11 days after access to the Northern Paddock, captive and wild immediate release animals were radiotracked for up to a total of 34 days. The area (ha) encompassing all diurnal locations of each animal was calculated using the 100 percentage minimum convex polygon method and compared between treatments using a randomisation test as well as a one way ANOVA on data transformed using ln+1. All bilbies were recaptured at one week, and seven to nine weeks, after release with delayed release animals also recaptured just prior to accessing the Northern Paddock (3 weeks). Statistical analyses were identical to those outlined previously for burrowing bettongs.

#### Stick-nest rats – captive-bred vs wild

Comparisons of wild and captive-bred stick-nest rats were completed in two experiments, a release of 6 wild and 19 captive-bred rats to the Main Paddock in 1999, and a release of 7 wild and 10 captive-bred rats into the Northern Paddock in 2003. The method of the initial release of stick-nest rats into the Main Paddock of the Arid Recovery Reserve in 1999 is summarised in Moseby et al. [33] and involved releasing animals immediately into the Main Paddock after dark. In this instance, wild rats were obtained from Reevesby Island, South Australia and transported to the release area in individual nest boxes by boat and then vehicle. Travel time and nest boxes were similar for both wild and captive-bred rats and all animals were released within 24 hours of capture.

The immediate release of 10 (5M, 5F) captive-bred rats into the Northern Paddock of the Arid Recovery Reserve occurred on 2 July 2003 at 20:00. Seven wild rats (2M, 5F) were transferred from the Main Paddock to the Northern Paddock between 5 and 27 July 2003. Due to the difficulty experienced capturing wild greater stick-nest rats, three were subadults.

All animals, captive-bred and wild, were weighed and ear tagged before immediate release at the same location. After release, rats were radiotracked daily and the vegetation species and density of vegetation cover for each diurnal shelter site recorded. Cover density was assessed by placing a 1.5 m pole painted with 5 cm black and white bands horizontally through the shrub at a height of approximately 30 cm. The number of black bands that could not be observed within the shrub was recorded as a percentage of the total number of bands known to be within the shrub. Animals were recaptured three weeks after release and re-weighed. If an animal was found dead, the carcass and surrounding area was carefully inspected for signs of predation.

At three weeks post release, movement data were analysed using the methods described previously and vegetation composition and cover were calculated within an area defined by the 90% minimum convex polygon of diurnal fixes of all rats. Two perpendicular 550 m line transects were placed through the diurnal use area and perennial shrub species and their cover density recorded at 10 m intervals. Results were used to compare the proportion of different shrub species and cover categories available as random shelter sites with actual diurnal shelter sites used by wild and captive rats. Data were compared using Chi-squared tests and contingency tables. Due to low sample sizes, data from individual rats were pooled within captive and wild treatments. We used Fisher's exact tests to determine whether there were any differences in rates of mortality between captive and wild animals as this test is reliable for any combination of sample sizes.

## Results

### Burrowing bettongs – immediate vs delayed

No burrowing bettongs died during the seven weeks of post release monitoring in the Northern Paddock. On the day following release the proportion of immediately released bettongs sheltering underground was significantly lower than delayed release animals (Fisher's exact test *P* = 0.01). All eight immediate release animals were found on the surface sheltering in thick vegetation on the morning after release and for up to 7 days after release. In comparison, only two of the six bettongs in the containment pen were found on the surface and all were underground by the second day. Three bettongs initially used the pre-excavated burrows and all dug their own burrows after a few days. Once the pen was opened, all of the delayed release bettongs were found in burrows on the day after they left the pen. Immediate and delayed bettongs were not found sharing burrows during the experiment.

**Figure 1:**
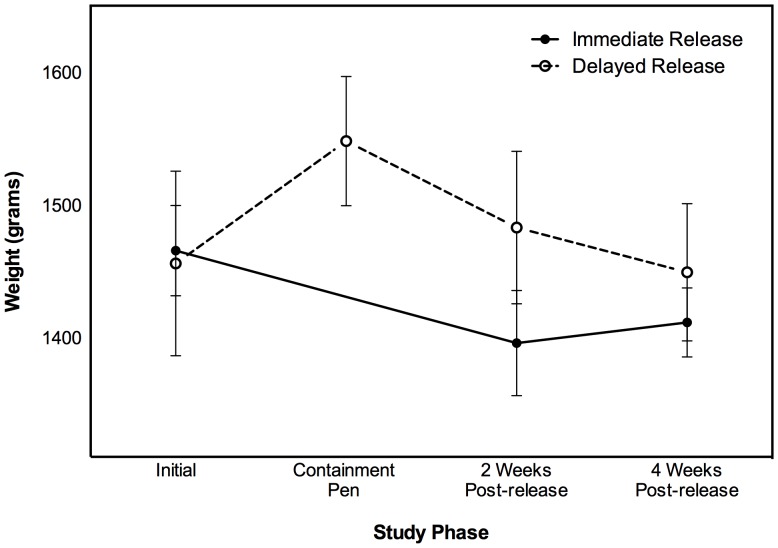
Weights of immediate (n = 8) and delayed (n = 6) release burrowing bettongs. The arrow indicates when release pen was first opened and delayed release animals allowed access to the Northern Paddock. Bars denote 1 standard error.

A linear mixed model conducted on weight data collected up until 4 weeks after access to the northern expansion found no difference between the weights of bettongs over time (F_2,28_ = 2.769, *P* = 0.08) or between treatments (delayed vs immediate F_1,14_ = 0.441, *P* = 0.52). However, there was a time by treatment interaction (F_2,28_ = 6.648, *P*<0.01) suggesting that the change in weight over time was different for delayed and immediate release animals. Delayed release animals gained weight in the pen before losing weight once the pen was opened (**Figure 1**). In comparison, immediate release animals lost weight initially before gaining weight. Our randomisation tests revealed a difference in weight between immediate and delayed release animals at two weeks after transfer to the Northern Paddock (*P* = 0.03). At four weeks after their transfer to the Northern Paddock, this difference was no longer evident (*P* = 0.24). No pouch young were recorded during post release monitoring but spoor counts [28] revealed that the population later expanded and remains extant in 2014. Medium term survival of both immediate and delayed release animals was high, with more than half (4 out of 6 delayed and 5 out of 8 immediate) of the released animals recaptured opportunistically between three months and three years after release (average delayed = 11 months, immediate = 15 months). A Fisher's exact test found no difference in the proportion of immediate and delayed release bettongs recaptured after three months (*P* = 1).

**Figure 2:**
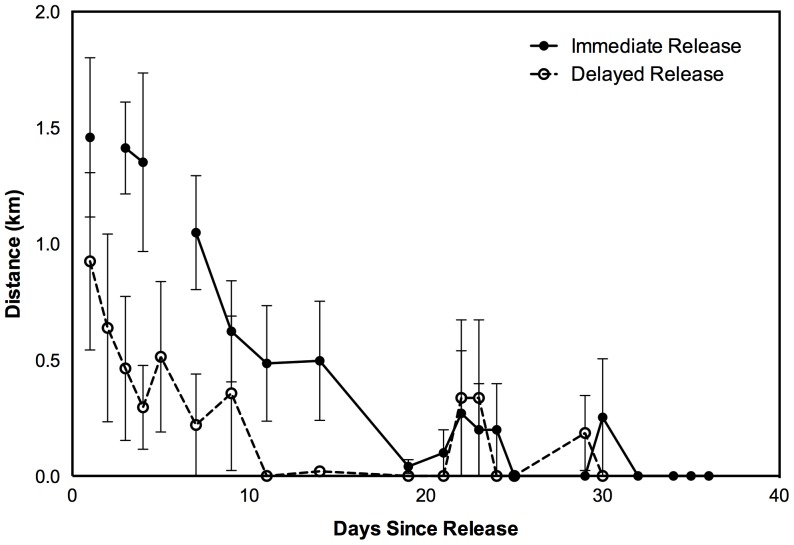
The distance between successive daily fixes for delayed and immediate release burrowing bettongs after access to the Northern Paddock. Delayed release bettongs were kept in a release pen for three weeks prior to release. Bars denote 1 standard error.

The average distance moved by immediate release bettongs on the night of release was 1.46 km (95% CI ±0.78) compared with 0.925 km (±0.75) for delayed release animals on the night after the pen was opened (**Figure 2**). However, due to the large variation between individuals both our t-test (t_12_ = −1.187, *P* = 0.26) and randomisation test (*P* = 0.25) revealed there was no difference between treatment groups. Movement between successive diurnal fixes declined to zero at around 11 days after the pen was opened for delayed release animals and approximately 19 days after release for immediate release bettongs (**Figure 2**).

After access to the Northern Paddock, the number of days until successive daily movements ceased and animals exhibited burrow fidelity was less in delayed release animals (3.6±2.1) than immediate release bettongs (13.1±4.8) (t_10.2_ = 3.457, *P*<0.01), (randomisation test *P* = 0.01). Two of the three delayed release females did not leave the release pen for more than a week after it was opened and their first move resulted in burrow fidelity.

### Greater bilbies - wild vs captive, immediate vs delayed

**Figure 3:**
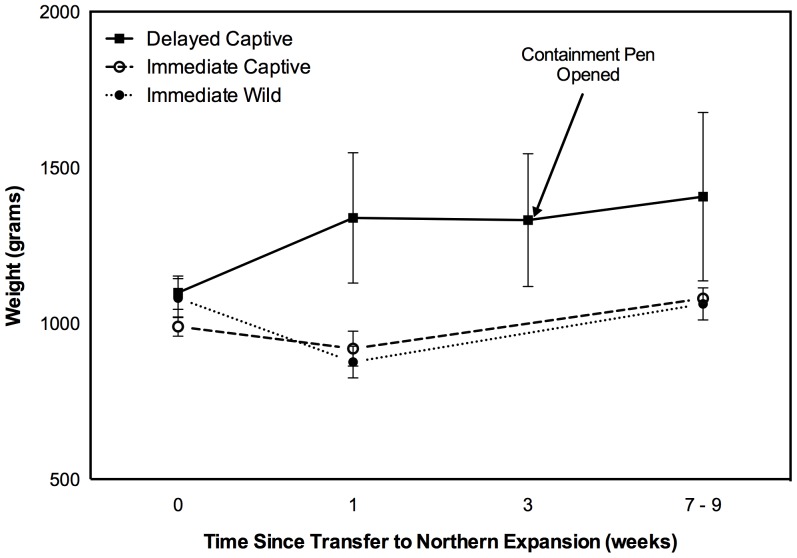
Average weights of delayed, immediate wild and immediate captive greater bilbies released into the Northern Paddock in 2003. Only three delayed and three immediate captive bilbies could be recaptured at seven to nine weeks for reweighing. Bars denote 1 standard error. Arrow indicates when release pen was opened.

No bilbies were known to have died after reintroduction into the Northern Paddock. Released bilbies were not known to use any pre-existing burrows in the northern expansion. Low sample sizes and difficulties recapturing bilbies rendered statistical comparisons of weight difficult. All released bilbies were a similar weight at the time of transfer to the Northern Paddock, but delayed release animals increased in weight after their introduction to the containment pen and after seven to eight days were heavier than immediate release wild and captive animals (randomisation tests *P*<0.01 and *P* = 0.02 respectively) (**Figure 3**). Delayed release animals maintained the weight increase after the containment pen was opened and immediate release animals had returned to their release weight seven to nine weeks after release such that there was no difference in weights between treatments (randomisation test *P* = 0.94, and *P* = 0.23 respectively). All female bilbies had pouch young when captured at seven to nine weeks after release.

**Figure 4:**
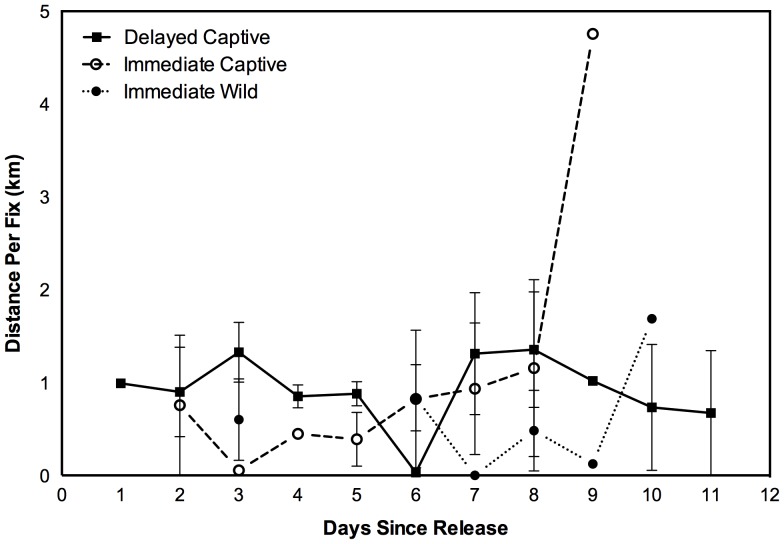
Average distances moved between radiotracking fixes for delayed and immediate release greater bilbies after release into the Northern Paddock. For delayed release animals, time since release refers to when the containment pen was opened and animals allowed access to the Northern Paddock. Bars denote 1 standard error, points without bars are single individuals.

Average movements between diurnal burrow fixes for the three treatments were typically less than 1.5 km each day (**Figure 4**), but missing data points meant that daily distances moved could not be compared between treatments. Within the first 11 days after release, each bilby was located on an average of 52% of these days (95% CI ±8.9%). The maximum distance moved from the release pen in the first 11 days after release was 4.5 km by an immediate release captive-bred male and the smallest distance was 0.29 km by an immediate release wild caught male. There was no difference in maximum distance moved between delayed and immediate release wild, and immediate captive bilbies (randomisation test *P* = 0.31 and *P* = 0.94 respectively; F_2_ = 0.337, *P* = 0.72) or between delayed and pooled immediate release bilbies (randomisation test *P* = 0.68; F_1_ = 0.743, *P* = 0.41).

There was no difference in the area encompassing the diurnal fixes of wild, immediate captive or delayed captive-bred bilbies (F_2_ = 0.045, *P* = 0.96) and our randomisation tests confirmed this between delayed release captive and immediate release wild (*P* = 0.74), immediate release captive and immediate release wild (*P* = 0.73), and delayed release captive and immediate release captive (*P* = 0.73) bilbies. Time until burrow fidelity was not compared, as bilbies are transient.

### Stick-nest rats - captive-bred vs wild

In 1999, two of six captive-bred rats (33.3%) and two of 19 wild caught rats (10.5%) died between six and 15 days after release. The cause of death was thought to be stress or malnutrition as intact carcasses were found with no signs of predation. Following the release into the Northern Paddock in 2003, four of ten captive-bred rats (40%) died and no deaths were recorded in wild rats (0%). Three captive-bred rats died within four days of release (two from predation by birds of prey and the third from unknown causes). The fourth rat died down a burrow 12 days after release after being observed on the surface lethargic and panting. Fisher's exact tests found no difference in rates of mortality between treatments in the 1999 or 2003 releases (*P* = 0.23 and 0.06 respectively). However, when the two releases were combined, captive-bred rats had higher rates of post release mortality than wild rats (*P* = 0.04).

In the 2003 release, the six captive-bred rats that survived all lost weight following release, averaging 326 g (95% CI ±24.6) at release, 278 g (±23.3) at three weeks and 292 g (±20.7) at five weeks post release. The single adult wild rat that was recaptured had lost 10 g in four weeks whilst the single recaptured subadult rat gained 115 g. At three weeks post release, wild rat shelter sites were further from the release site (729 m, 95% CI ±716.5) than captive rats (65 m±33.9) (t_11_ = 2.664, *P* = 0.02) (randomisation test *P* = 0.02). Two of the wild rats settled 1 549 m and 2 581 m from the release site.

There were 223 diurnal locations recorded for captive rats and 121 for wild rats over the first month after the 2003 release. Burrowing bettong warrens were used on 18 occasions (8%) by captive rats and 10 occasions (12%) by wild rats and were excluded from the analysis of plant species and cover selection. There was a difference in plant species selected for shelter sites between the two treatments (*x^2^* = 25.970, d.f. = 3, n = 316, *P*<0.001). Captive-bred rats selected a greater proportion of sandhill canegrass (*Zygochloa paradoxa*), sandhill wattle and ruby saltbush (*Enchylaena tomentosa*) than expected according to chance (*x^2^* = 41.719, d.f. = 4, *P*<0.001, n = 205) and wild rats selected a greater proportion of sandhill canegrass, the plant species with the thickest cover (*x^2^* = 55.43, d.f. = 4, *P*<0.001, n = 111). Similarly, both captive-bred (*x^2^* = 169.269, d.f. = 4, n = 205, *P*<0.001) and wild (*x^2^* = 107.37, d.f. = 4, n = 111, *P*<0.001) rats selected denser than average shelter sites and wild rats chose denser sites than captive-bred rats (*x^2^* = 56.208, d.f. = 4, n = 316, *P*<0.001). Wild rats chose shelter sites with the thickest cover (80–100%) and captive rats utilised a wider range of cover densities including sparser shrubs.

## Discussion

Differences in post release survival, movements, weight dynamics and settling times were recorded between different species and release strategies. Our results support the inconsistent outcomes obtained from past reviews on this topic [3]–[7] and suggest that broad reviews of release strategies do not appropriately inform future reintroduction programs. Instead, what is needed is a categorical classification of ‘under what scenario is a particular reintroduction protocol likely to succeed?’ We propose that life history and behavioural traits such as shelter dependence, site fidelity, sociality, and ranging behaviour are some of the species characters that determine the appropriate reintroduction strategy. These intrinsic factors are influenced by critical extrinsic factors such as whether the release site is bounded or unbounded, and whether predation risk is high or low. The influence of some of these intrinsic and extrinsic factors are herewith discussed both individually, using results from the present study and previous research, and in a combined predictive model which could be tested and refined using experimentation and manipulation in a variety of species and situations.

### Release sites

The nature of the release site, whether bounded or unbounded, has significant implications for the choice of release protocol and behaviour of released animals. Our study could not detect any differences in the scale of movements between different treatment groups of released animals and this is likely due to our bounded release site constraining potential long range movements. For example, the maximum distance from one corner of the Northern Paddock to another is approximately 9 km. Immediate releases of bettongs in unbounded areas outside the Arid Recovery Reserve in 2008 resulted in movements of up to 18 km from the release point [33] and several male burrowing bettongs released to Herisson Prong in Western Australia were recorded moving more than 10 km and up to 21 km from the release site [20]. Comparative studies on other species of mammal have found similar large-scale movements of animals released immediately into large, unbounded release areas [12], [47].

The post release movement of animals is an important consideration for the success of a reintroduction program. There is evidence that dispersing individuals have higher mortality rates than non-dispersers and males that disperse large distances from the release site are unlikely to contribute to the reintroduced population [14], [15], [48], [49]. In unbounded release sites, containment of wide ranging species on-site prior to release may improve the outcomes of reintroduction programs by helping to retain animals closer to the release site where additional supportive measures such as exotic predator control can be intensified.

### Predation risk

Predation is a major cause of failure in reintroduction programs [6]. We detected no difference in rates of mortality between captive and wild bilbies, a species most vulnerable to mammalian predators that were absent from the reserve. However, captive-bred greater stick-nest rats suffered higher rates of mortality than did wild-bred animals, a large proportion of which were taken by birds of prey. Stick-nest rats are smaller than bilbies and more vulnerable to aerial predators [44] and captive-bred stick-nest rats chose poorer shelter sites that were likely to leave them more exposed to aerial predation. Similarly, a reintroduction trial of five numbats (*Myrmecobius fasciatus*) (a diurnal species) into the Arid Recovery reserve in 2005 also failed due to predation by birds of prey [50]. Hence, even at the same release site, the level of predation risk varies between species due to different physical, behavioural or life history traits. The choice of whether to use wild or captive-bred stock must consider the specific predation risk of the species in question, with species at greater risk of predation likely to benefit from the use of wild stock. As predation risk is species-specific, the choice of captive or wild stock may be of little consequence when predation risk is low.

For the immediate vs delayed experiments, our results did not suggest either protocol led to greater overall survival. As in our study, many of the reintroduction studies have found no difference between delayed and immediate release animals where conducted in situations of low predation risk [23], [30], [51]. Successful immediate releases have often been onto islands or fenced reserves [20], [21], [33], [39], [52] and may partly explain why Short [7] found immediately released animals in Australian reintroductions had greater survival than delayed release animals.

### Shelter investment and site fidelity

The inter-specific differences we observed in response to release protocols may have partly reflected the level of dependence on specific shelter sites. Both bettongs and greater stick-nest rats invest significant energy into building permanent shelters that can be utilised over several generations. Both depend upon their shelters for protection from predators, and the microclimate generated within shelters also protects them from environmental extremes, to which greater stick-nest rats are particularly vulnerable [53]. Poor shelter selection by captive-bred rats likely contributed to the higher rates of mortality and predation. Likewise, a greater proportion of immediately released bettongs were found above ground on the first day after release and they also took longer to settle in a single location. Species with higher site fidelity may simply be more disrupted by translocation, travelling larger distances in an attempt to return to their home sites, and becoming exposed to increased stress and predation risk. In these cases, delayed releases and/or the provision of artificial shelters may be beneficial.

Transient species that do not invest heavily in building permanent burrows or nests may find reintroductions less stressful than sedentary species that exhibit higher site fidelity. Bilbies are expert diggers, move burrows regularly and hence can quickly establish a new burrow after release [39]. Therefore, there appeared to be little difference in the success of immediate, delayed, wild or captive release protocols for bilbies. Similarly, no differences in movement or condition were detected between immediate and delayed release mala (*Lagorchestes hirsutus*) [23] or mountain sheep (*Ovis canadensis*) [54], both of which are relatively transient and do not invest significant energy in building shelters.

### Sociality

Social species such as bettongs may also find reintroductions more stressful than solitary species like the bilby and could benefit from delayed releases. Our results showed that bettongs might settle within one area, and form new social groupings faster when a delayed release protocol is employed. In France, female rabbits survived better when acclimatised using a delayed release [55] with improvements attributed to sex-specific social behaviour. Kleiman [14] suggested that in social species, dispersal after release may be partly because translocated animals often lack familiarity with individuals at the release site. Wimberger et al. [56] reported that a reintroduction of the gregarious rock hyrax (*Procavia capensis*) in South Africa failed due to high dispersal, and suggested delayed releases of family groups would improve reintroduction outcomes. Shier [57] used delayed releases of family groups of black tailed prairie dogs (*Cynomys ludovicianus*) to improve reintroduction success and found higher survival than non-family groups. Reintroduction success of the social European ground squirrel (*Spermophilus citellus*) was increased when delayed release pens were used to prevent panic dispersal, establish a new social order and adjust to new food resources [16]. The use of release pens in social, sedentary species that spend significant time establishing permanent shelters may assist reintroduction success by keeping family groups intact, allowing time for communal burrow or den establishment and reducing stress.

### Ranging Behaviour

The ranging behaviour of a species should also be considered when developing release strategies. For example, stick-nest rats have small home ranges and high nest fidelity. In our study, they did not travel far from the release site suggesting that the use of containment pens to constrain movements in this species may be unnecessary. Conversely, a range of release strategies such as containment and the provision of water or supplementary food may be important for keeping wide-ranging species within release areas [29].

### Framework for future experiments

We found that post release survival, movement, weight loss and time until site fidelity differed between release strategies and species, highlighting the importance of developing species and location-specific release strategies. Survival of reintroduced individuals is a key prerequisite to success and the likelihood of survival of reintroduced populations is a function of predation risk, site characteristics, and specific life history and behavioural traits. These are the primary elements that should be used to develop successful, individually-tailored release strategies. All characteristics must be acknowledged, and we suggest the following future experimentation framework for identifying how these factors interact with post release reintroduction outcomes.

**Figure 5:**
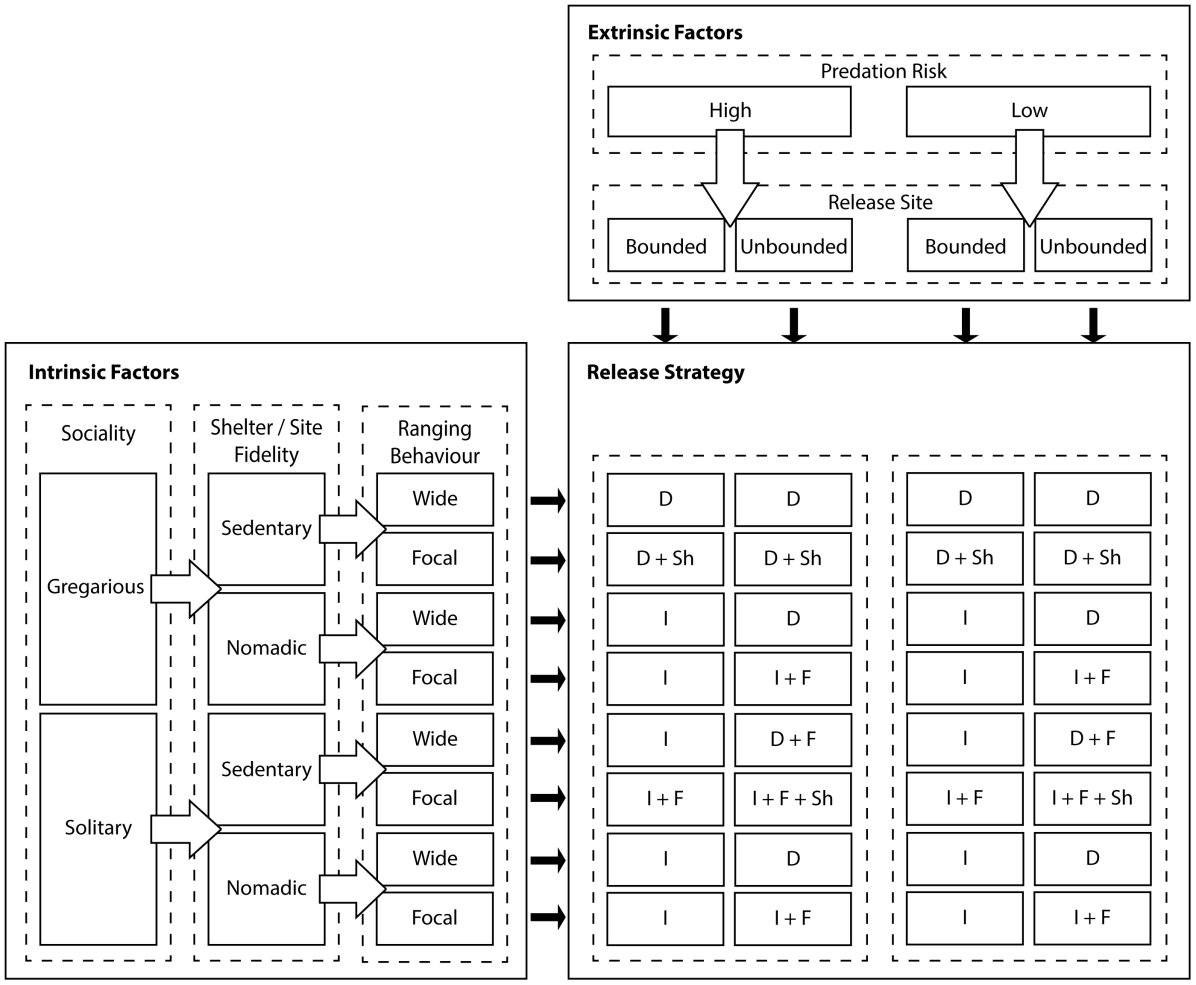
A predictive model based on current and previous studies which could be used as a basis for hypothesis testing regarding which release strategies are the most suitable for a given species reintroduction, depending on the site characteristics. The model could be tested against both captive-bred, and wild groups of release animals. Key to release strategies: I = immediate release, D = delayed release, F = supplementary food, Sh = supplementary shelter.

Firstly, individual experiments need to include careful apriori analysis of which extrinsic and intrinsic factors are likely to influence reintroduction success based on the study species and location in question. We suggest classification of sites and species under the following categories: predation risk (high or low); release site (bounded or unbounded); sociality (gregarious or solitary); shelter site fidelity (nomadic or sedentary) and range behaviour (wide-ranging or focal). These factors can be used to develop a predictive model outlining which reintroduction protocols may assist projects with particular species and release site characteristics (e.g. **Figure 5**). These identified factors are not exclusive, and we expect that additional considerations such as disease risk may also need to be incorporated into the model under different scenarios.

Secondly, experiments then need to be designed to test these hypotheses and build on reintroduction theory. An informative assessment of the advantages and disadvantages of various release protocols will require meta-analysis of dozens of repeat experiments using species with different behavioural and life history traits. In order for meta-analysis to occur, practitioners need to collect and collate similar data to enable meaningful comparisons. We see the following data as being the minimum requirement for such trials: post release movement distances; rates of mortality; rates of predation; time until settlement (for sedentary species); distance settled from release site; and weight dynamics (for at least three months following release).

Finally, publication of individual reintroduction experiments with small sample sizes needs to be encouraged in order to facilitate meta-analyses and appropriate reviews. Currently, the results of many studies are unavailable in the scientific literature partly because scientific journals are reluctant to publish studies with low statistical power. This lack of replication is an inherent part of reintroduction biology due to the fact that rare species only allow for low numbers of individuals to be released. Opportunities for publishing reintroduction outcomes are available through initiatives such as the IUCN Re-introduction Specialist Group [58] but more stringent and standardised reporting requirements are required to ensure data are useful for future meta-analysis. Critically, when assessing the success of different release strategies, global reviews need to ensure that behavioural traits, predation risk and release site attributes are included as co-variates and that the various components of delayed releases are analysed separately.
